# Supplementary material for: Sequence comparison of the mitochondrial genomes of Plesionika species (Caridea: Pandalidae), gene rearrangement and phylogenetic relationships of Caridea
Source: PeerJ. 2024 May 22;12:e17314. doi: 10.7717/peerj.17314 (PMC11127644; doi:10.7717/peerj.17314)
Supplement: Supplemental Information 1 [file peerj-12-17314-s001.docx]

**Supplementary Information**

**Table S1.** List of species analyzed in this study and their GenBank accession numbers，and three newly sequenced *Plesionika* species were marked with✽.

| Sufamily | Family | Species | Size(bp) | GenBank | References |
| --- | --- | --- | --- | --- | --- |
| Alpheoidea | Alpheidae | *Alpheus digitalis* | 15,700 | NC_014883 | *Qian et al., (2011)* |
|  |  | *Alpheus hoplocheles* | 15,735 | NC_038068 | Unpublished |
|  |  | *Alpheus inopinatus* | 15,789 | NC_041151 | Unpublished |
|  |  | *Alpheus japonicus* | 16,619 | NC_038116 | Unpublished |
|  |  | *Alpheus bellulus* | 15,738 | MH796167 | *Wang et al., (2019)* |
|  |  | *Alpheus lobidens* | 15,735 | KP276147 | *Tan et al., (2017)* |
|  |  | *Alpheus randalli* | 15,676 | MH796168 | *Wang et al., (2019)* |
|  |  | *Synalpheus microneptunus* | 15,603 | NC_047307 | Unpublished |
|  |  | *Leptalpheus forceps* | 15,463 | MN732884 | Unpublished |
|  | Lysmatidae | *Lysmata amboinensis* | 16,735 | NC_050676 | Unpublished |
|  |  | *Lysmata boggessi* | 17,345 | NC_064049 | Unpublished |
|  |  | *Lysmata debelius* | 16,757 | NC_060421 | Unpublished |
|  |  | *Lysmata vittata* | 22,003 | NC_049878 | *Zhu et al., (2021)* |
|  |  | *Exhippolysmata ensirostris* | 16,350 | MK681888 | *Ye et al., (2021)* |
|  | Thoridae | *Thor amboinensis* | 15,553 | NC_051930 | Unpublished |
|  |  | *Lebbeus groenlandicus* | 17,399 | NC_045223 | Unpublished |
|  | Hippolytidae | *Saron marmoratus* | 16,330 | NC_050677 | Unpublished |
| Atyoidea | Atyidae | *Stygiocaris lancifera* | 15,787 | NC_035404 | Unpublished |
|  |  | *Stygiocaris stylifera* | 15,812 | NC_035411 | Unpublished |
|  |  | *Typhlatya arfeae* | 15,887 | NC_035410 | Unpublished |
|  |  | *Typhlatya consobrina* | 15,758 | NC_035407 | Unpublished |
|  |  | *Typhlatya dzilamensis* | 15,892 | NC_035408 | Unpublished |
|  |  | *Typhlatya galapagensis* | 16,430 | NC_035402 | Unpublished |
|  |  | *Typhlatya garciai* | 15,318 | NC_035409 | Unpublished |
|  |  | *Typhlatya iliffei* | 15,926 | NC_035401 | Unpublished |
|  |  | *Typhlatya miravetensis* | 15,865 | NC_036335 | Unpublished |
|  |  | *Typhlatya mitchelli* | 15,814 | NC_035403 | Unpublished |
|  |  | *Typhlatya monae* | 16,007 | NC_035405 | Unpublished |
|  |  | *Typhlatya pearsei* | 15,798 | NC_035400 | Unpublished |
|  |  | *Typhlatya taina* | 15,790 | NC_035399 | Unpublished |
|  |  | *Typhlopatsa pauliani* | 15,824 | NC_035406 | Unpublished |
|  |  | *Caridina gracilipes* | 15,550 | NC_024751 | *Xu et al., (2016)* |
|  |  | *Caridina indistincta* | 15,461 | NC_039593 | Unpublished |
|  |  | *Caridina longshan* | 15,556 | OP177695 | Unpublished |
|  |  | *Caridina multidentata* | 15,825 | NC_038067 | Unpublished |
|  |  | *Neocaridina davidi* | 15,564 | MN418055 | Unpublished |
|  |  | *Paratya australiensis* | 15,990 | NC_027603 | *Gan et al., (2016)* |
|  |  | *Halocaridina rubra* | 16,065 | NC_008413 | Unpublished |
|  |  | *Halocaridinides fowleri* | 15,977 | NC_035412 | *Jurado*-Rivera *et al., (2017)* |
|  |  | *Neocaridina denticulata* | 15,561 | NC_023823 | *Yu et al., (2014)* |
| Palaemonoidea | Palaemonidae | *Palaemon adspersus* | 15,736 | NC_050168 | Unpublished |
|  |  | *Palaemon annandalei* | 15,718 | NC_038117 | Unpublished |
|  |  | *Palaemon capensis* | 15,925 | NC_039373 | Unpublished |
|  |  | *Palaemon gravieri* | 15,740 | NC_029240 | *Kim et al. (2015b)* |
|  |  | *Palaemon serratus* | 15,758 | NC_050266 | Unpublished |
|  |  | *Palaemon serenus* | 15,967 | NC_027601 | *Gan et al. (2016b)* |
|  |  | *Palaemon varians* | 14,889 | MT340090 | Unpublished |
|  |  | *Palaemon elegans* | 15,650 | MT340089 | *González-Castellano et al., (2020)* |
|  |  | *Palaemon modestus* | 15,736 | MF687349 | Unpublished |
|  |  | *Macrobrachium nipponense* | 15,806 | NC_015073 | *Ma et al. (2011)* |
|  |  | *Macrobrachium rosenbergii* | 15,772 | NC_006880 | *Miller et al., (2005)* |
|  |  | *Macrobrachium bullatum* | 15,774 | KM978918 | *Tan et al., (2017)* |
|  |  | *Hymenocera picta* | 15,786 | NC_039631 | *Sung et al., (2018)* |
| Bresilioidea | Alvinocarididae | *Alvinocaris chelys* | 15,910 | NC_018778 | *Yang et al. (2012)* |
|  |  | *Alvinocaris longirostris* | 16,022 | NC_042497 | *Yang et al. (2013)* |
|  |  | *Alvinocaris kexueae* | 15,864 | MH714459 | Unpublished |
|  |  | *Rimicaris paulexa* | 15,909 | NC_051948 | Unpublished |
|  |  | *Mirocaris indica* | 15,922 | NC_054368 | Unpublished |
|  |  | *Nautilocaris saintlaurentae* | 15,928 | NC_021971 | *Kim et al. (2015a)* |
|  |  | *Opaepele loihi* | 15,905 | NC_020311 | *Yang et al. (2013)* |
|  |  | *Rimicaris exoculata* | 15,902 | NC_027116 | *Yu et al. (2015)* |
|  |  | *Shinkaicaris leurokolos* | 15,903 | NC_037487 | Unpublished |
|  |  | *Manuscaris liui* | 15,903 | MH714461 | Unpublished |
|  |  | *Rimicaris kairei* | 15,900 | NC_020310 | *Yang et al. (2013)* |
|  |  | *Rimicaris variabilis* | 15,909 | MN419306 | *Lee et al., (2019)* |
| Pandaloidea | Pandalidae | *Bitias brevis* | 15,891 | NC_040856 | *Sun et al., (2020)* |
|  |  | *Chlorotocus crassicornis* | 15,935 | NC_035828 | *Kim et al., (2019)* |
|  |  | *Heterocarpus ensifer* | 15,939 | NC_040855 | *Sun et al., (2020)* |
|  |  | *Heterocarpus sibogae* | 16,036 | NC_071946 | Unpublished |
|  |  | *Pandalus borealis* | 15,956 | LC341266 | Unpublished |
|  |  | *Pandalus prensor* | 17,194 | MW091549 | Unpublished |
|  |  | *Parapandalus sp.* | 16,037 | MH714458 | Unpublished |
|  |  | *Plesionika edwardsii* | 15,956 | OP087601 | Unpublished |
|  |  | *Plesionika sindoi* | 15,908 | MH714453 | Unpublished |
|  |  | ✽*Plesionika ortmanni* | 15,908 | OP650932 | This study |
|  |  | ✽*Plesionika izumiae* | 16,074 | OP650933 | This study |
|  |  | ✽*Plesionika lophotes* | 15,933 | OP650934 | This study |
| Oplophoroidea | Acanthephyridae | *Notostomus gibbosus* | 17,590 | NC_059935 | *Yuan et al., (2021)* |
|  |  | *Acanthephyra smithi* | 17,165 | MH714455 | Unpublished |
|  | Oplophoridae | *Oplophorus spinosus* | 17,346 | NC_059714 | *Wang et al., (2021)* |
|  |  | *Oplophorus typus* | 16,883 | MH714457 | Unpublished |
| Nematocarcinoidea | Nematocarcinidae | *Nematocarcinus gracilis* | 15,919 | MH714456 | Unpublished |

**Table S2.** Annotation of three newly sequenced *Plesionika* species mitochondrial genome.

| Gene | Strand | Location | | | | | | Size (bp) | | | Anticodon |
| --- | --- | --- | --- | --- | --- | --- | --- | --- | --- | --- | --- |
|  |  | *P. ortmanni* | | *P. izumiae* | | *P. lophotes* | | *P. ortmanni* | *P. izumiae* | *P. lophotes* |  |
|  |  | form | to | form | to | form | to |  |  |  |  |
| *COI* | + | 1 | 1533 | 1 | 1554 | 1 | 1554 | 1533 | 1554 | 1554 | / |
| *trnL2* | + | 1529 | 1594 | 1555 | 1613 | 1550 | 1619 | 66 | 59 | 70 | TAA |
| *CO**II* | + | 1599 | 2286 | 1628 | 2314 | 1626 | 2312 | 688 | 687 | 687 | / |
| *trnD* | + | 2355 | 2420 | 2424 | 2492 | 2332 | 2395 | 66 | 69 | 64 | GTC |
| *trnK* | + | 2287 | 2354 | 2355 | 2415 | 2402 | 2470 | 68 | 61 | 69 | TTT |
| *ATP8* | + | 2421 | 2579 | 2521 | 2679 | 2503 | 2661 | 159 | 159 | 159 | / |
| *ATP6* | + | 2573 | 3247 | 2673 | 3347 | 2655 | 3329 | 675 | 675 | 675 | / |
| *COIII* | + | 3247 | 4035 | 3350 | 4138 | 3332 | 4120 | 789 | 789 | 789 | / |
| *trnG* | + | 4042 | 4106 | 4143 | 4209 | 4124 | 4190 | 65 | 67 | 67 | TCC |
| *ND3* | + | 4107 | 4460 | 4222 | 4563 | 4200 | 4544 | 354 | 342 | 345 | / |
| *trnA* | + | 4460 | 4524 | 4577 | 4639 | 4557 | 4619 | 65 | 63 | 63 | TGC |
| *trnR* | + | 4526 | 4592 | 4643 | 4709 | 4623 | 4689 | 67 | 67 | 67 | TCG |
| *trnN* | + | 4595 | 4660 | 4716 | 4782 | 4697 | 4763 | 66 | 67 | 67 | GTT |
| *trnS1* | + | 4661 | 4727 | 4782 | 4850 | 4764 | 4832 | 67 | 69 | 69 | TCT |
| *trnE* | + | 4728 | 4795 | 4857 | 4924 | 4839 | 4906 | 68 | 68 | 68 | TTC |
| *trnF* | − | 4794 | 4861 | 4925 | 4990 | 4907 | 4971 | 68 | 66 | 65 | GAA |
| *ND5* | − | 4861 | 6576 | 4971 | 6689 | 4977 | 6620 | 1716 | 1719 | 1644 | / |
| *trnH* | − | 6589 | 6652 | 6708 | 6773 | 6690 | 6753 | 64 | 66 | 64 | GTG |
| *ND4* | − | 6653 | 7991 | 6774 | 8112 | 6760 | 8092 | 1339 | 1339 | 1333 | / |
| *ND4L* | − | 7985 | 8284 | 8112 | 8405 | 8092 | 8385 | 300 | 294 | 294 | / |
| *trnT* | + | 8286 | 8350 | 8407 | 8473 | 8387 | 8453 | 65 | 67 | 67 | TGT |
| *trnP* | − | 8351 | 8414 | 8474 | 8538 | 8454 | 8517 | 64 | 65 | 64 | TGG |
| *ND6* | + | 8417 | 8932 | 8541 | 9059 | 8547 | 9038 | 516 | 519 | 492 | / |
| *Cytb* | + | 8932 | 10,113 | 9059 | 10,210 | 9038 | 10,189 | 1182 | 1152 | 1152 | / |
| *trnS2* | + | 10,067 | 10,138 | 10,194 | 10,263 | 10,173 | 10,242 | 72 | 70 | 70 | TGA |
| *ND1* | − | 10,160 | 11,098 | 10,353 | 11,291 | 10,318 | 11,262 | 939 | 939 | 945 | / |
| *trnL1* | − | 11,148 | 11,213 | 11,335 | 11,403 | 11,300 | 11,368 | 66 | 69 | 69 | TAG |
| *16S* | − | 11,174 | 12,534 | 11,380 | 12,707 | 11,345 | 12,666 | 1361 | 1328 | 1322 | / |
| *trnV* | − | 12,537 | 12,603 | 12,732 | 12,802 | 12,690 | 12,760 | 67 | 71 | 71 | TAC |
| *12S* | − | 12,606 | 13,408 | 12,805 | 13,615 | 12,763 | 13,574 | 803 | 811 | 812 | / |
| *CR* | + | 13,694 | 14,166 | 13,861 | 13,928 | 13,581 | 13,684 | 472 | 68 | 104 | / |
| *trnI* | + | 14,479 | 14,545 | 14,564 | 14,630 | 14,518 | 14,585 | 67 | 67 | 68 | GAT |
| *trnQ* | − | 14,561 | 14,628 | 14,738 | 14,805 | 14,604 | 14,671 | 68 | 68 | 68 | TTG |
| *trnM* | + | 14,628 | 14,695 | 14,806 | 14,874 | 14,671 | 14,738 | 68 | 69 | 68 | CAT |
| *ND2* | + | 14,696 | 15,697 | 14,908 | 15,873 | 14,772 | 15,737 | 1002 | 966 | 966 | / |
| *trnW* | + | 15,700 | 15,768 | 15,872 | 15,939 | 15,736 | 15,803 | 69 | 68 | 68 | TCA |
| *trnC* | − | 15,768 | 15,834 | 15,940 | 16,003 | 15,803 | 15,866 | 67 | 64 | 64 | GCA |
| *trnY* | − | 15,835 | 15,902 | 16,005 | 16,069 | 15,868 | 15,932 | 68 | 65 | 65 | GTA |

**Table S3.** Start and stop codons for PCGs of three *Plesionika* species.

| Gene | Start Codon | | | Stop Codon | | |
| --- | --- | --- | --- | --- | --- | --- |
|  | *P. ortmanni* | *P. izumiae* | *P. lophotes* | *P. ortmanni* | *P. izumiae* | *P. lophotes* |
| *CO**I* | ACG | ATG | ATG | TAA | TAG | TAG |
| *COII* | ATG | ATG | ATG | T(AA) | TAA | TAA |
| *ATP8* | ATC | ATG | ATA | TAA | TAA | TAA |
| *ATP6* | ATG | ATG | ATG | TAA | TAA | TAA |
| *COIII* | ATG | ATG | ATG | TAG | TAA | TAA |
| *ND3* | ATT | ATA | ATC | TAG | TAA | TAA |
| *ND5* | ATT | ATG | ATT | TAA | TAG | T(AA) |
| *ND4* | ATG | ATG | ATG | T(AA) | T(AA) | T(AA) |
| ND*4L* | ATG | ATG | ATG | TAA | TAA | TAA |
| *ND6* | ATT | ATT | ATT | TAA | TAA | TAA |
| *Cytb* | ATG | ATG | ATG | TAA | TAG | TAG |
| *ND1* | ATA | ATT | ATA | TAA | TAG | TAA |
| *ND2* | ATT | ATA | ATA | TAA | TAA | TAA |

**Table S4.** Codon number and relative synonymous codon usage of three newly sequenced *Plesionika* species mitochondrial genome.

| Codon | Count | Rscu | Codon | Count | Rscu | Codon | Count | Rscu | Codon | Count | Rscu |
| --- | --- | --- | --- | --- | --- | --- | --- | --- | --- | --- | --- |
| *P. ortmanni* |  |  |  |  |  |  |  |  |  |  |  |
| UUU(F) | 211 | 1.24 | UCU(S) | 111 | 1.55 | UAU(Y) | 159 | 1.28 | UGU(C) | 53 | 1.22 |
| UUC(F) | 129 | 0.76 | UCC(S) | 59 | 0.83 | UAC(Y) | 89 | 0.72 | UGC(C) | 34 | 0.78 |
| UUA(L) | 179 | 1.72 | UCA(S) | 85 | 1.19 | UAA(*) | 184 | 1.37 | UGA(W) | 72 | 1.43 |
| UUG(L) | 59 | 0.57 | UCG(S) | 32 | 0.45 | UAG(*) | 84 | 0.63 | UGG(W) | 29 | 0.57 |
| CUU(L) | 130 | 1.25 | CCU(P) | 114 | 1.42 | CAU(H) | 100 | 1.04 | CGU(R) | 22 | 0.83 |
| CUC(L) | 88 | 0.85 | CCC(P) | 97 | 1.21 | CAC(H) | 93 | 0.96 | CGC(R) | 24 | 0.91 |
| CUA(L) | 124 | 1.19 | CCA(P) | 84 | 1.05 | CAA(Q) | 105 | 1.32 | CGA(R) | 46 | 1.74 |
| CUG(L) | 43 | 0.41 | CCG(P) | 26 | 0.32 | CAG(Q) | 54 | 0.68 | CGG(R) | 14 | 0.53 |
| AUU(I) | 198 | 1.22 | ACU(T) | 134 | 1.63 | AAU(N) | 186 | 1.21 | AGU(S) | 58 | 0.81 |
| AUC(I) | 126 | 0.78 | ACC(T) | 80 | 0.97 | AAC(N) | 121 | 0.79 | AGC(S) | 90 | 1.26 |
| AUA(M) | 165 | 1.54 | ACA(T) | 92 | 1.12 | AAA(K) | 224 | 1.4 | AGA(S) | 88 | 1.23 |
| AUG(M) | 49 | 0.46 | ACG(T) | 23 | 0.28 | AAG(K) | 96 | 0.6 | AGG(S) | 49 | 0.69 |
| GUU(V) | 74 | 1.39 | GCU(A) | 59 | 1.26 | GAU(D) | 62 | 1.18 | GGU(G) | 51 | 1.19 |
| GUC(V) | 37 | 0.69 | GCC(A) | 65 | 1.39 | GAC(D) | 43 | 0.82 | GGC(G) | 34 | 0.80 |
| GUA(V) | 71 | 1.33 | GCA(A) | 54 | 1.16 | GAA(E) | 81 | 1.42 | GGA(G) | 53 | 1.24 |
| GUG(V) | 31 | 0.58 | GCG(A) | 9 | 0.19 | GAG(E) | 33 | 0.58 | GGG(G) | 33 | 0.77 |
| *P. izumiae* |  |  |  |  |  |  |  |  |  |  |  |
| UUU(F) | 218 | 1.36 | UCU(S) | 111 | 1.51 | UAU(Y) | 173 | 1.24 | UGU(C) | 40 | 0.95 |
| UUC(F) | 102 | 0.64 | UCC(S) | 75 | 1.02 | UAC(Y) | 107 | 0.76 | UGC(C) | 44 | 1.05 |
| UUA(L) | 208 | 1.98 | UCA(S) | 96 | 1.31 | UAA(*) | 199 | 1.57 | UGA(W) | 71 | 1.37 |
| UUG(L) | 47 | 0.45 | UCG(S) | 22 | 0.30 | UAG(*) | 55 | 0.43 | UGG(W) | 33 | 0.63 |
| CUU(L) | 157 | 1.49 | CCU(P) | 131 | 1.51 | CAU(H) | 74 | 0.97 | CGU(R) | 21 | 1.02 |
| CUC(L) | 58 | 0.55 | CCC(P) | 96 | 1.11 | CAC(H) | 78 | 1.03 | CGC(R) | 21 | 1.02 |
| CUA(L) | 126 | 1.2 | CCA(P) | 93 | 1.07 | CAA(Q) | 112 | 1.54 | CGA(R) | 27 | 1.32 |
| CUG(L) | 35 | 0.33 | CCG(P) | 27 | 0.31 | CAG(Q) | 33 | 0.46 | CGG(R) | 13 | 0.63 |
| AUU(I) | 229 | 1.41 | ACU(T) | 127 | 1.38 | AAU(N) | 196 | 1.14 | AGU(S) | 65 | 0.88 |
| AUC(I) | 96 | 0.59 | ACC(T) | 95 | 1.03 | AAC(N) | 147 | 0.86 | AGC(S) | 66 | 0.90 |
| AUA(M) | 206 | 1.63 | ACA(T) | 133 | 1.44 | AAA(K) | 297 | 1.58 | AGA(S) | 106 | 1.44 |
| AUG(M) | 46 | 0.37 | ACG(T) | 14 | 0.15 | AAG(K) | 80 | 0.42 | AGG(S) | 47 | 0.64 |
| GUU(V) | 62 | 1.47 | GCU(A) | 71 | 1.47 | GAU(D) | 60 | 1.12 | GGU(G) | 32 | 0.93 |
| GUC(V) | 27 | 0.64 | GCC(A) | 56 | 1.16 | GAC(D) | 47 | 0.88 | GGC(G) | 27 | 0.79 |
| GUA(V) | 62 | 1.47 | GCA(A) | 57 | 1.18 | GAA(E) | 71 | 1.43 | GGA(G) | 56 | 1.64 |
| GUG(V) | 18 | 0.43 | GCG(A) | 9 | 0.19 | GAG(E) | 28 | 0.57 | GGG(G) | 22 | 0.64 |
| *P. lophotes* |  |  |  |  |  |  |  |  |  |  |  |
| UUU(F) | 213 | 1.34 | UCU(S) | 118 | 1.73 | UAU(Y) | 186 | 1.22 | UGU(C) | 42 | 1.09 |
| UUC(F) | 105 | 0.66 | UCC(S) | 67 | 0.98 | UAC(Y) | 118 | 0.78 | UGC(C) | 35 | 0.91 |
| UUA(L) | 246 | 2.14 | UCA(S) | 96 | 1.41 | UAA(*) | 256 | 1.46 | UGA(W) | 55 | 1.31 |
| UUG(L) | 72 | 0.63 | UCG(S) | 14 | 0.21 | UAG(*) | 95 | 0.54 | UGG(W) | 29 | 0.69 |
| CUU(L) | 130 | 1.13 | CCU(P) | 136 | 1.63 | CAU(H) | 97 | 1.13 | CGU(R) | 9 | 0.67 |
| CUC(L) | 50 | 0.44 | CCC(P) | 88 | 1.06 | CAC(H) | 75 | 0.87 | CGC(R) | 9 | 0.67 |
| CUA(L) | 144 | 1.25 | CCA(P) | 84 | 1.01 | CAA(Q) | 132 | 1.43 | CGA(R) | 29 | 2.15 |
| CUG(L) | 47 | 0.41 | CCG(P) | 25 | 0.30 | CAG(Q) | 52 | 0.57 | CGG(R) | 7 | 0.52 |
| AUU(I) | 199 | 1.38 | ACU(T) | 107 | 1.30 | AAU(N) | 163 | 1.14 | AGU(S) | 58 | 0.85 |
| AUC(I) | 89 | 0.62 | ACC(T) | 88 | 1.07 | AAC(N) | 122 | 0.86 | AGC(S) | 67 | 0.98 |
| AUA(M) | 195 | 1.59 | ACA(T) | 112 | 1.36 | AAA(K) | 325 | 1.55 | AGA(S) | 86 | 1.26 |
| AUG(M) | 50 | 0.41 | ACG(T) | 22 | 0.27 | AAG(K) | 94 | 0.45 | AGG(S) | 40 | 0.59 |
| GUU(V) | 52 | 1.41 | GCU(A) | 50 | 1.36 | GAU(D) | 54 | 1.05 | GGU(G) | 40 | 1.24 |
| GUC(V) | 23 | 0.62 | GCC(A) | 42 | 1.14 | GAC(D) | 49 | 0.95 | GGC(G) | 30 | 0.93 |
| GUA(V) | 55 | 1.49 | GCA(A) | 46 | 1.25 | GAA(E) | 74 | 1.4 | GGA(G) | 45 | 1.4 |
| GUG(V) | 18 | 0.49 | GCG(A) | 9 | 0.24 | GAG(E) | 32 | 0.6 | GGG(G) | 14 | 0.43 |

**References**

**Gan HY, Gan HM, Lee YP, Austin CM. 2016.** The complete mitogenome of the Australian freshwater shrimp *Paratya australiensis* Kemp, 1917 (Crustacea: Decapoda: Atyidae). *Mitochondrial DNA* **27(5):**3157–3158

DOI 10.3109/19401736.2015.1007312.

**Gan HY, Gan HM, Tan MH, Lee YP, Austin CM. 2016b.** The complete mitogenome of the hermit crab *Clibanarius infraspinatus* (Hilgendorf, 1869), (Crustacea; Decapoda; Diogenidae) – a new gene order for the Decapoda. *Mitochondrial DNA* **27:**4099–4100 DOI 10.3109/19401736.2014.1003862.

**González-Castellano I, Pons J, González-Ortegón E, Martínez-Lage A. 2020.** Mitogenome phylogenetics in the genus *Palaemon* (Crustacea: Decapoda) sheds light on species crypticism in the rockpool shrimp *P. elegans*. *PLoS One* **15(8):**e0237037 DOI 10.1371/journal.pone.0237037.

**Jurado-Rivera JA, Pons J, Alvarez F, Botello A, Humphreys WF, Page TJ, Iliffe TM, Willassen E, Meland K, Juan C, Jaume D. 2017.** Phylogenetic evidence that both ancient vicariance and dispersal have contributed to the biogeographic patterns of anchialine cave shrimps. *Scientific Reports* **7(1):**2852

DOI 10.1038/s41598-017-03107-y.

**Kim G, Alam MJ, Kim HW, Andriyono S. 2019.** Complete mitochondrial genome of green shrimp, *Chlorotocus crassicornis* (Crustacea: Decapoda: Pandalidae) in Korean water. *Mitochondrial DNA B* **4(2):**2206–2207.

DOI 10.1080/23802359.2019.1624633.

**Kim SJ, Pak SJ, Ju SJ. 2015a.** Mitochondrial genome of the hydrothermal vent shrimp *Nautilocaris saintlaurentae* (Crustacea: Caridea: Alvinocarididae). *Mitochondrial DNA* 26:127–128 DOI 10.3109/19401736.2013.815169.

**Kim S-T, Kim G, Yoon T-H, Kim A-R, Kim HJ, Kim H-W. 2015b.** Complete mitochondrial genome of *Palaemon gravieri* (Yu, 1930) (Crustacea: Decapoda: Palaemonidae). *Mitochondrial DNA* 1–2 DOI 10.3109/19401736.2015.1118078.

**Lee WK, Kim SJ, Ju SJ, Hou BK.** **2019.** Complete mitochondrial genome of the hydrothermal vent shrimp *Rimicaris variabilis* (Decapoda: Caridea: Alvinocarididae) from the North Fiji basin. *Mitochondrial DNA* **4(2):**3475–3476 DOI 10.1080/23802359.2019.1674717.

**Ma K, Feng J, Lin J, Li J. 2011.** The complete mitochondrial genome of *Macrobrachium nipponense*. *Gene* **487:**160–165

DOI 10.1016/j.gene.2011.07.017.

**Miller AD, Murphy NP, Burridge CP, Austin CM. 2005.** Complete mitochondrial DNA sequences of the decapod crustaceans *Pseudocarcinus gigas* (Menippidae) and *Macrobrachium rosenbergii* (Palaemonidae). *Marine Biotechnology* **7(4):**339–349. DOI 10.1007/s10126-004-4077-8.

**Qian G, Zhao Q, Wang AN, Zhu LIN, Zhou K, Sun H. 2011.** Two new decapod (Crustacea, Malacostraca) complete mitochondrial genomes: bearings on the phylogenetic relationships within the Decapoda. *Zoological Journal of the Linnean Society* **162(3):**471–481 DOI 10.1111/j.1096-3642.2010.00686.x.

**Shen X, Sun MA, Wu Z, Tian M, Cheng H, Zhao F, Meng X. 2009.** The complete mitochondrial genome of the ridgetail white prawn *Exopalaemon carinicauda* Holthuis, 1950 (Crustacean: Decapoda: Palaemonidae) revealed a novel rearrangement of tRNA genes. *Gene* **437:**1–8 DOI 10.1016/j.gene.2009.02.014.

**Sung CH, Cheng CC, Lu JK, Wang LJ. 2018.** The complete mitochondrial genome of *Hymenocera picta* (Malacostraca: Decapoda: Hymenoceridae). *Mitochondrial DNA* **3(2):**790–791 DOI 10.1080/23802359.2018.1481779.

**Sun SE, Cheng J, Sun S, Sha Z. 2020.** Complete mitochondrial genomes of two deep-sea pandalid shrimps, *Heterocarpus ensifer* and *Bitias brevis*: insights into the phylogenetic position of Pandalidae (Decapoda: Caridea). *Journal of Oceanology and Limnology* **38(3):**816–825 [DOI 10.1007/s00343-019-9040-x](https://doi.org/10.1007/s00343-019-9040-x).

**Tan MH, Gan HM, Lee YP, Poore GC, Austin CM.** **2017.** Digging deeper: new gene order rearrangements and distinct patterns of codons usage in mitochondrial genomes among shrimps from the Axiidea, Gebiidea and Caridea (Crustacea: Decapoda). *PeerJ* **5:**e2982 DOI 10.7717/peerj.2982.

**Qian GG, Zhao Q, Wang A, Zhu L, Zhou KY, Sun HY. 2011.** Two new decapod (Crustacea, Malacostraca) complete mitochondrial genomes: bearings on the phylogenetic relationships within the Decapoda. *Zoological Journal of the Linnean Society* **162(3):**71–481, DOI 1096-3642.2010.00686.x.

**Wang XT, Shi WG, Li YX, Zhang XL, Xu QZ. 2021.**The complete mitochondrial genome of *Oplophorus spinosus* (Brullé, 1839) (Caridea, Oplophoridae). *Mitochondrial DNA* **6(5):**1597–1598

DOI 10.1080/23802359.2021.1914221.

**Wang, Q., Wang, Z.Q., Tang, D., Xu, X.Y., Tao, Y.T., Ji, C.Y., & Wang, Z.F. (2019).** Characterization and comparison of the mitochondrial genomes from two Alpheidae species and insights into the phylogeny of Caridea. *Genomics* **112(1):**65–70 DOI 10.1016/j.ygeno.2019.08.013.

**Xu GC, Du FK, Du, Nie** **ZJ, Xu P, Gu RB. 2016.** Complete mitochondrial genome of *Caridina nilotica gracilipes* *Mitochondrial DNA* **27(2):**1249–1250

DOI 10.3109/19401736.2014.945541.

**Yang C-H, Tsang LM, Chu KH, Chan T-Y. 2012.** Complete mitogenome of the deep-sea hydrothermal vent shrimp *Alvinocaris chelys* Komai and Chan, 2010 (Decapoda: Caridea: Alvinocarididae). *Mitochondrial DNA* **23:**417–419

DOI 10.3109/19401736.2012.710212.

**Yang JS, Lu B, Chen DF, Yu YQ, Yang F, Nagasawa H, Tsuchida S, Fujiwara Y, Yang WJ. 2013.** When did decapods invade hydrothermal vents? Clues from the Western Pacific and Indian Oceans. *Molecular biology and evolution* **30:**305–309 DOI 10.1093/molbev/mss224.

**Ye YY, Miao J, Guo YH, Gong L, Guo BY. 2021.** The first mitochondrial genome of the genus *Exhippolysmata* (Decapoda: Caridea: Lysmatidae), with gene rearrangements and phylogenetic associations in Caridea. *Scientific Reports* **11(1):**1–13 DOI 10.1038/s41598-021-93946-7.

**Yuan C, Shi W, Dong Y, Li Y, Xu Q.** **2021.** The first complete mitochondrial genome of *Notostomus gibbosus* (Caridea, Acanthephyridae). *Mitochondrial DNA* **6(7):**2050–2051 DOI 10.1080/23802359.2021.1942259.

**Yu YQ, Liu XL, Li HW, Lu B, Fan YP, Yang JS. 2015.** The complete mitogenome of the Atlantic hydrothermal vent shrimp *Rimicaris exoculata* Williams & Rona 1986 (Crustacea: Decapoda: Alvinocarididae). *Mitochondrial DNA* 1–3 DOI 10.3109/19401736.2015.1007291.

**Yu YQ, Yang WJ, Yang JS. 2014.** The complete mitogenome of the Chinese swamp shrimp *Neocaridina denticulata* sinensis Kemp 1918 (Crustacea: Decapoda: Atyidae). *Mitochondrial DNA* **25:**204–205

DOI 10.3109/19401736.2013.796465.

**Zhu L, Zhu Z, Zhu L,** **Wang D, Wang J, Lin Q. 2021.** The complete mitogenome of *Lysmata vittata* (Crustacea: Decapoda: Hippolytidae) and its phylogenetic position in Decapoda. *Cold Spring Harbor Laboratory* **16(11):**e0255547

DOI 10.1101/2021.08.04.455109.
